# Endobacteria Have a Negative Effect on the Virulence of *Metarhizium*

**DOI:** 10.3390/jof11110813

**Published:** 2025-11-16

**Authors:** Aida Gabriela Mora-Acebedo, Isay Ruíz Aguilar, Azul Martínez-Vázquez, Iván Horacio Piña-Torres, Arelí Durón Castellanos, Zulia Fernandina Nieves-López, Jorge Contreras-Garduño, Gloria Angélica González-Hernández, Israel Enrique Padilla-Guerrero, Juan Carlos Torres-Guzmán

**Affiliations:** 1 Departamento de Biología, División de Ciencias Naturales y Exactas, Universidad de Guanajuato, Noria Alta s/n, Guanajuato 36050, Estado de Guanajuato, Mexico; ag.moraacebedo@ugto.mx (A.G.M.-A.); isay1994@hotmail.com (I.R.A.); madr.martinez@ugto.mx (A.M.-V.); ih.pinatorres@gmail.com (I.H.P.-T.); areli_duron@ugto.mx (A.D.C.); gonzang@ugto.mx (G.A.G.-H.); ie.padillaguerrero@ugto.mx (I.E.P.-G.); 2 Cinvestav Unidad Irapuato, Km 9.6 Libramiento Norte, Carretera Irapuato-León, Irapuato 36824, Estado de Guanajuato, Mexico; zulia.nieves@uan.edu.mx; 3 Escuela Nacional de Estudios Superiores Unidad Morelia, Universidad Autónoma de México (UNAM), Antigua carretera a Pátzcuaro No. 8701, Morelia 58190, Michoacán, Mexico; jcg@enesmorelia.unam.mx

**Keywords:** *Metarhizium*, endobacterium, mycopesticides, biological pest control, *Bacillus subtilis*

## Abstract

Most organisms are associated with microorganisms, which influence their behavior during their life cycles. Fungi are no exception; they interact with plants, viruses, and bacteria in various environments, forming complex communities. These associations can occur externally around the mycelia and internally within the hyphae. Fungi can harbor bacteria, mycoviruses, and other fungi within their hyphae. Some endobacteria (EB) have been shown to alter fungal host function, development, and interactions with other organisms. Most fungi that host endobacteria (EB) are plant-associated. Although members of the genus *Metarhizium* are among the most abundant fungi isolated from soils, their associated EB have not been sufficiently studied. Endobacteria were recently detected in *M. bibiondarum* and *M. anisopliae*; however, the biological roles of these bacteria in the different *Metarhizium* life cycles remain unknown. In this study, *Metarhizium* strains were isolated from the rhizosphere and *Phyllophaga* spp. *Bacillus subtilis* was identified as an endobacterium, and its influence on the physiology of *Metarhizium* and entomopathogenic capacity was studied. Our analysis revealed that EB have a negative impact on the virulence of *Metarhizium* against *Galleria mellonella* and *Tenebrio molitor.*

## 1. Introduction

Almost all organisms are associated with microorganisms during their life cycles, and some of these associations significantly influence their behavior. Symbiosis, the interaction between two or more species during development, is an example of this interaction. Based on the cost–benefit dynamics among the involved species, various symbiotic interactions occur, including parasitism, mutualism, and commensalism [1]. Fungi are not an exception; they interact with viruses and bacteria in various environments, forming complex communities that can influence their behavior. These associations can occur externally around the mycelia and internally within the hyphae [2]. Bacteria-like organisms living symbiotically inside the *Endogone* fungal hyphae have been reported since 1970 [3]. Filamentous fungi can harbor bacteria, mycoviruses, and other fungi within their hyphae [4]. Some endobacteria (EB) have been shown to alter fungal host function, development, and interactions with other organisms [2,4,5]. EB have been identified in different phyla of fungi, including Mucormycota, Ascomycota, and Basidiomycota [6]. Robinson et al. [7] screened 702 phylogenetically diverse fungal isolates representing 366 genera for potential bacterial associates, detecting bacterial associates in 88% of the analyzed fungal isolates [7]. Members of Proteobacteria, Actinobacteria, and Firmicutes were the most commonly associated with fungal isolates. The authors suggest that bacteria–fungi associations are the rule rather than the exception [7].

The presence of EB in fungi holds significant implications for the biology of the host fungus; for instance, one of the earliest models of fungus–endobacterium symbiosis was described for the mycorrhizal fungus *Gigaspora margarita* and the *Burkholderia*-related endobacterium *Candidatus* Glomeribacter gigasporarum (*Ca*Cg) [8,9]. The presence of the endobacterium *Ca*Cg affects the expression of genes involved in growth, development, and transport in its fungal host [10]. The endobacterium increases the fungal sporulation and increases the fungal bioenergetic capacity [10]. The authors suggested that endobacterium potentially increases the fungal innate immune response [10].

Another example of the importance of the fungus–endobacteria association is the plant pathogenic fungus *Rhizopus microsporus*, which causes rice seedling blight. In this fungus, the rhizoxin, a phytotoxin that binds the β-tubulin, inhibits mitosis and induces cell cycle arrest; it is biosynthesized by EB in the genus *Burkholderia* [11]. In the same sense, *R. microsporus* contains the *Ralstonia pickettii* bacterial endosymbiont, which is required for virulence in zebrafish and mouse models and suppresses the growth of the amoeba *Dictyostelium discoideum* [12]. The endobacterium *B. rhizoxinica* is not only involved in *R. microsporus* virulence but also in spore formation, which is dependent on the presence of EB. The reproduction of the fungus is dependent on the EB, which, in turn, facilitates the formation of the toxin [13]. These EB, previously classified as *Burkholderia* and then *Paraburkholderia* [14], now form the novel genus *Mycetohabitans* within *Burkholderia* sensu lato [15]. Other fungi also contain EB; for example, in *Ustilago maydis,* the presence of a bacterial endosymbiont related to *Bacillus pumilus* with the capacity to fix nitrogen has been reported [16,17].

Most of the fungi that host EB are plant-associated, including arbuscular and ectomycorrhizal fungi, plant pathogens, saprotrophic fungi, and endophytes within plant tissues [2]. These EB may contribute to plant growth and disease resistance [5].

More than 90% of land plant species have symbiotic relationships with fungi [18]. Members of the genus *Metarhizium* are among the most abundant fungi isolated from soils [19,20]. *Metarhizium* has been isolated from soils and infected insects on all continents except Antarctica [21], and can colonize various environments, including forests, savannas, swamps, coastal zones, and deserts [22]. *Metarhizium* has a multifunctional lifestyle [23], being an insect-pathogenic fungus that can infect and kill more than 200 insect species, including those in at least seven orders [21]. *Metarhizium* plays a crucial ecological role and is closely associated with the plant rhizosphere. *Metarhizium* endophytically colonizes the roots of various plants, transferring insect-derived nitrogen to the plant host [24,25], and receives carbon that the plant fixes through photosynthesis [26]. This symbiotic interaction provides multiple benefits, promoting plant growth [27,28,29,30], improving salt tolerance [31,32], and protecting plants against foliar pests and plant-pathogenic fungi [33]. *Metarhizium* is also an antagonist of root pathogens, such as *Fusarium* [34], and promotes plant defense against invertebrate pests [35,36]. *Metarhizium* is not an obligate plant symbiont and can survive as a saprophyte [37].

Genomic analysis has shown that *Metarhizium* spp. is more closely related to endophytes and plant pathogens than to arthropod pathogens [23]. In this sense, the ancestors of entomopathogenic species were probably root-colonizing saprophytes that subsequently acquired genes for insect pathogenesis [38]. According to these antecedents, the *Metarhizium’s* ability to switch between different lifestyles in soil could be influenced by bacteria present in the rhizosphere. *Metarhizium*–bacteria interactions have not been intensively studied, and bacterial interactions may influence *Metarhizium* behavior as an entomopathogen, plant growth promoter, or during plant endophytic colonization. Recently, the endobacteria *Pelomonas paraquae* were detected within the hyphae and conidia of *M. bibiondarum* and *M. anisopliae* [39]. However, the biological role of these bacteria in the different *Metarhizium* lifestyles remains unknown. In this study, strains of *Metarhizium* were isolated from the rhizosphere and *Phyllophaga* spp. *Bacillus subtilis* was identified as an endobacterium, and its influence on the physiology and entomopathogenic capacity of *Metarhizium* was studied.

## 2. Materials and Methods

### 2.1. Fungal Isolates

Soil samples were collected in the “Las Siete Luminarias” nature reserve (20°18′46.2″ N 101°12′10.4″ W located in Valle de Santiago, Guanajuato, México, from a depth of approximately 10 cm, using a sterile hand shovel, and stored in polypropylene bags and transported to the laboratory. For each sample, 1 g of soil and 10 mL of 0.01% Triton™ X-100 (Sigma-Aldrich^®^, Saint Louis, MO, USA) were mixed in a 15 mL Falcon tube using a vortex mixer. Serial dilutions from 10^−1^ to 10^−7^ were prepared for each sample; 100 μL of each dilution was inoculated in a non-selective medium (Potato Dextrose Agar, PDA (Bioxon^®^, Cuautitlán Izcalli, México)) and incubated at 26 °C with a photoperiod of 16 h light/8 h darkness for 10–15 days. Single colonies that morphologically resembled *Metarhizium* were transferred to new PDA plates and incubated under the same conditions for 10 days, until conidiation occurred. Conidia were collected using a sterile toothpick and placed in an Eppendorf tube containing 1 mL of 0.01% Triton™ X-100 (Sigma-Aldrich^®^) homogenized in a vortex mixer for 1 min and then counted using a Neubauer chamber (Hausser Scientific^®^ Horsham, PA, USA). The concentration was adjusted to 1000 conidia/mL. A 50 μL volume of this dilution was inoculated on new PDA plates and incubated at 26 °C with a photoperiod of 16 h light/8 h darkness for ten days. Single colonies were selected and grown in minimal media, M-100 plates (containing 0.2% NH_4_NO_3_, 1% dextrose, 0.3% KH_2_PO_4_, and 2% 50× salt stock solution (25 g MgSO_4_, 0.09 g ZnSO_4_, 0.05 g FeSO_4_, 0.015 g MnSO4, and 0.02 g CuSO_4_)) incubated at 26 °C with photoperiod of 16 h light/8 h darkness for ten days until conidiation. Conidia were collected by scraping the colony, and the hyphae were removed by filtration through a sheet of sterile cheesecloth. The conidia were then washed three times by centrifugation and suspended in a sterile 0.01% Triton™ X-100 (Sigma-Aldrich^®^) solution. Additionally, larvae of *Phyllophaga* spp. were collected from soil obtained from “Puruagua,” Jerécuaro, Guanajuato, México (20°04′46.6″ N 100°27′14.6″ W). Each larva was collected manually and individually confined in a plastic container filled with sterilized soil. Plastic containers were incubated at room temperature. Dead larvae with visible *Metarhizium* colonization were collected, and conidia were recovered using the same protocol described for isolating *Metarhizium* from soil samples.

### 2.2. Identification of Metarhizium Isolates

*Metarhizium* isolates were initially identified based on morphological criteria and subsequently confirmed by molecular characterization using the gene sequence of the translation elongation factor 1-alpha (TEF1-α) [40]. The *Metarhizium* isolates were grown at 28 °C in Sabouraud Dextrose Medium (SDM, Bioxon^®^, Cuautitlán Izcalli, México) for 24 h. Mycelia were recovered through filtration, and DNA was isolated using standard protocols [41]. A fragment of the TEF1-α gene was amplified by PCR using the primers EFT1 (5′-ATGGGTARGGAAGACAAGAC-3′) and EFT2 (5′-GGAAGTACCAGTGATCATGTT-3′). PCR conditions were 95 °C for 5 min, 40 cycles at 95 °C for 20 s, 55 °C for 1 min, 72 °C for 1.5 min, and an additional cycle at 72 °C for 5 min. The PCR product was purified using the GenElute™ Gel Extraction kit (Sigma-Aldrich^®^), cloned into the pGEM^®^-T Easy Vector (Promega Corporation, Madison, WI, USA), and sequenced (Elim Biopharm, Inc., Hayward, CA, USA). The TEF1-α gene sequences were aligned against available representative sequences of the genus *Metarhizium* using MAFFT (https://www.ebi.ac.uk/Tools/msa/mafft/) (accessed on 1 November 2025).

### 2.3. Bacteria-Free Metarhizium

To eliminate associated bacteria, conidia from the *Metarhizium* ES37 and PPH1 strains were successively inoculated in M-100 medium plates containing ciprofloxacin (50 µg·µL^−1^) and cefotaxime (100 µg·µL^−1^) antibiotics at 26 °C with a photoperiod of 16 h light/8 h darkness for ten days, until conidiation occurred. Conidia were collected by scraping the colony, the hyphae were removed by filtration through a sheet of sterile cheesecloth, and the conidia were washed three times by centrifugation and suspended in a sterile 0.01% Triton™ X-100 (Sigma-Aldrich^®^) solution. This procedure was repeated at least six times.

### 2.4. Endobacteria Isolation

Endobacteria were isolated following a previously described protocol [11], with some modifications. A stock solution of conidia from the *Metarhizium* ES37 and PPH1 strains containing 1 × 10^6^ conidia·mL^−1^ was prepared. From this solution, 100 μL was inoculated in PDA Petri plates containing a cellophane film. The plates were incubated at 26 °C for 48 h in dark conditions to promote mycelial growth. Mycelia were recovered from the cellophane film and placed in a porcelain mortar containing 300 μL of glass beads and 1 mL of 1M NaCl. The mycelia were crushed with a porcelain pestle, and the disrupted cells were centrifuged at 19,315× *g* for 3 min. Subsequently, 75 μL of the supernatant was transferred to five different media. The Eppendorf tubes were again centrifuged at 19,315× *g* for 10 min, and 20 μL of the supernatant was transferred to five different media: 1. Nutrient agar (BD Bioxon^®^, Cuautitlán Izcalli, México); 2. Yeast Mannitol Agar, YMA (yeast extract (BD Bioxon^®^, Cuautitlán Izcalli, México), 1.5 g·L^−1^ mannitol (Sigma-Aldrich^®^, Saint Louis, MO, USA), 10 g·L^−1^ MgSO_4_ (Karal^®^, León, Gto. México), 0.20 K_2_HPO_4_ g·L^−1^ (Karal^®^), 0.20 g·L^−1^ FeCl_3_ (Karal^®^), 1 mL^−1^ bacteriological agar (BD Bioxon^®^), and Congo red (Sigma-Aldrich^®^, Saint Louis, MO, USA), pH 7, 10 mL·L^−1^); 3. YMA without Congo red; 4. Luria–Bertani (BD Bioxon^®^, Cuautitlán Izcalli, México); and 5. Actinomycete selective medium (20 g soybean flour, 5 g yeast extract (BD Bioxon^®^), and 20 g mannitol (Sigma-Aldrich^®^)). Samples were incubated at 30 °C for 24 h. Bacterial isolates were then selected and purified by repeated subculturing and maintained in the corresponding media.

### 2.5. Identification of Bacterial Isolates

Bacterial colonies were grown overnight at 30 °C in YMA medium without Congo red, and the cells were collected by centrifugation. Bacterial cells were identified morphologically by Gram staining. Bacterial cells were recovered and crushed in a porcelain mortar with liquid nitrogen. The obtained powder was resuspended in 400 mL of NTES (0.1 M NaCl, 10 mM Tris–HCl (pH 8.0), 1 mM EDTA, and 1% SDS), and DNA extraction was performed using the phenol–chloroform technique [41]. For molecular identification, the 16S rDNA was amplified using JumpStart™ Taq ReadyMix™ (Sigma-Aldrich^®^, Saint Louis, MO, USA) and three pairs of universal primers: (1) 63F (CAGGCCTAACACATGCAAGTC) and M1387R (GGGCGGWGTGTACAAGRC); (2) 27F (AGAGTTTGATCMTGGCTCAG) and 1492R (TACGGYTACCTTGTTACGACTT); (3) 27F (AGAGTTTGATCMTGGCTCAG) and R1494 (CTACGGRTACCTTGTTGTTACGAC) [42,43]. PCR conditions were 95 °C for 1 min, 32 cycles at 95 °C for 20 s, 55 °C for 45 s, 72 °C for 2 min, and an additional cycle at 72 °C for 5 min. PCR products of the expected size (approximately 1.5 Kbp) were purified using the GenElute™ Gel Extraction kit (Sigma-Aldrich^®^), cloned into the pGEM™-T Easy Vector (Promega Corporation, Madison, WI, USA), and sequenced (Elim Biopharm, Inc., USA, Hayward, CA, USA).

### 2.6. Bacterial DNA Extraction and Sequencing

To extract bacterial genomic DNA from the *B. subtilis* 1E strain, bacterial cultures were grown in liquid minimal medium (0.67% BD Yeast Nitrogen Base, 1% BD dextrose) to a concentration of 6 × 10^7^ cells·mL^−1^, and cells were harvested by centrifugation in the early log phase of growth. Total bacterial DNA was extracted using the DNeasy Blood & Tissue Kit (Qiagen, cat. no. 69504, Redwood City, CA, USA) according to the manufacturer’s instructions, which included a pretreatment step for Gram-positive bacteria. DNA integrity was verified by electrophoresis, and DNA concentration was measured using a NanoDrop 2000c spectrophotometer (Thermo Fischer Scientific, Waltham, MA, USA). The bacterial genomic DNA from *B. subtilis* 1E was sequenced using the whole-genome sequencing and assembly of genomic DNA service offered by Plasmidsaurus (https://plasmidsaurus.com) (accessed 3 March 2025), employing the hybrid option. This option combines long-read sequencing using Oxford Nanopore Technology with Illumina bacterial genome sequencing to polish the Oxford Nanopore reads. The sequencing results for *B. subtilis* 1E are presented in Table S1.

### 2.7. Comparative Genomic Analysis

To investigate the pangenome structure of *B. subtilis* 1E, we performed pangenome analysis using PPanGGOLiN v1.1.126, using the default parameters [44]. The study included the genomes of *B. subtilis* 1E (NCBI accession number CP196825) and the reference *B. subtilis* strains BSP1 (GCF_000321395.1) and 168 (GCF_000009045.1), both retrieved from the NCBI RefSeq database. This approach allowed us to identify gene families shared by all strains, as well as those exclusive to either endosymbiotic or reference genomes, providing insights into their genomic diversities and potential functional differences. Following the comparative analysis, the singletons in *B. subtilis* 1E were annotated using eggNOG-mapper v2.1.13 [45]. The annotation and function prediction were performed using different databases: GeneMarkS, Gene Ontology (GO), Kyoto Encyclopedia of Genes and Genomes (KEGG), and Clusters of Orthologous Groups (COG).

### 2.8. Localization of Endobacteria Using Microscopy

Endobacterial detection using microscopy was performed according to a previously described protocol [11], using the green-fluorescent nucleic acid stain SYTO^®^9 (Thermo Fisher Scientific, Waltham, MA, USA). The mycelia of a growing culture of *Metarhizium* strains were transferred to an Eppendorf tube containing 0.5 mL of 0.85% NaCl. An aliquot (10 μL) was placed on a microscope slide and stained with 0.5 μL of SYTO^®^9 (5 μM). The samples were incubated in the dark for 30 min and analyzed using a laser scanning confocal microscope (ZEISS LSM 700, Jena, Germany).

### 2.9. Conidial Yield

To determine the conidial yield, 1000 conidia from *Metarhizium* ES37 and PPH1 strains and their bacteria-free derivatives (*Metarhizium* ES37-2 and PPH1-1 strains) were inoculated in the center of Petri dishes containing M-100 media or Potato Dextrose Agar (PDA). The Petri plates were incubated at 28 °C for 10 days (M-100) or 15 days (PDA) with a photoperiod of 16 h of light and 8 h of darkness, or in complete darkness. After incubation, the colony diameter was measured, and the conidia were collected and resuspended in 1 mL of 0.01% Triton™ X-100 (Sigma-Aldrich^®^). Conidia concentration was determined using a Neubauer chamber under a microscope. The experiments were performed three times, with three replicates per experiment.

### 2.10. Conidia Germination Assay

To determine whether the presence of endobacteria in *Metarhizium* affects conidia germination, 1 × 10^3^ conidia were inoculated in a sterile microscope glass slide containing 2.5 mL of M-100 or PDA medium. Slides were incubated at 28 °C for 12, 18, and 24 h. At least 300 cells were counted under the microscope for each time point, and the percentage of germlings and ungerminated conidia was determined. A germling was defined as a germ tube equal in length to the width of the conidia. The experiments were performed three times, with three replicates per experiment.

### 2.11. Insect Bioassays

Fresh conidia of *Metarhizium* strains with endobacteria (ES37 and PPH1) and endobacteria-free *Metarhizium* strains (ES37-2 and PPH1-1) were used in bioassays against *Galleria mellonella* larvae. For each strain, sixty larvae were inoculated by immersion for 5 s with conidia suspended in 0.01% Triton™ X-100 (Sigma-Aldrich^®^) at a dilution of 1 × 10^8^ conidia·mL^−1^. An additional 60 larvae were inoculated with 0.01% Triton™ X-100 (Sigma-Aldrich) and served as a control. Infection was monitored every 24 h. Larvae were fed an artificial diet daily. The experiments were performed in four independent assays.

Fresh conidia of a *Metarhizium* strain with endobacteria (ES37) and an endobacteria-free *Metarhizium* strain (ES37-2) were used in bioassays against *Tenebrio molitor* larvae. All larvae were bred in plastic cages (47 × 26 × 12 cm). Wheat bran and cornmeal (1:1 ratio) were provided ad libitum as food, and apples and oranges were provided twice a week. A photoperiod of 12 h of light and 12 h of darkness, a temperature of 27 ± 2 °C, and a relative humidity of 45 ± 5% were maintained in the colony. Size was controlled (1.5–1.8 cm) in all experiments. A previously described experimental protocol was used [46]. Larvae (*n* = 300) were injected with a 1 μL suspension of PBS–Tween^®^ 80 (0.01%) (Sigma-Aldrich^®^, Saint Louis, MO, USA) containing three different conidia concentrations (5, 50, and 100 conidia) of the *Metarhizium* strain containing endobacteria (ES37) and endobacteria-free *Metarhizium* strain (ES37-2). PBS–Tween^®^ 80 (0.01%) (Sigma-Aldrich^®^) was used as a control. Injections were performed through the pleural intersegmental membrane between the sixth and seventh abdominal segments using sterilized micro syringes (10 μL Hamilton^®^ syringe, Hamilton Company, Reno, NV, USA). Larvae were individually separated in plastic plates with 12 wells (Corning, Monterrey, NL, México), and food was provided as described above. Survival was recorded every day for 10 days, and the cause of death was corroborated. The experiments were performed in four independent assays.

### 2.12. Phylogenetic Analyses

Phylogenetic analyses were performed using the 5′ sequence of the *TEF1*α gene from 63 *Metarhizium* isolates, employing the statistical Neighbor-Joining method in MEGA12 [47]. The phylogenetic tree was constructed using Bayesian inference with Mr. Bayes v3.2.6 software and the GTR model with an invariant gamma distribution range, with a bootstrap of 1000.

### 2.13. Statistical Analysis

Statistical analyses were performed using the GraphPad Prism v10.6.1 software (Boston, MA, USA). A *p*-value < 0.0001 is considered statistically significant. Data normality was assessed using the Shapiro–Wilk test. Conidiation results were analyzed using one-way ANOVA and the Kruskal–Wallis test, as appropriate. For germination assays, the unpaired *t*-test and the Mann–Whitney U test were applied, depending on whether the assumptions of normality were met. For survival analyses, the Kaplan–Meier method was used to generate the survival curves, and the log-rank (Mantel–Cox) test was applied to compare survival distributions between groups.

## 3. Results

### 3.1. Associated Endobacteria Can Be Identified in Native Metarhizium Strains

To investigate the presence of endobacteria in *Metarhizium*, strains from the rhizosphere and larvae of *Phyllopagha* spp. were isolated in two different regions of Guanajuato, México. The isolates were cultured in media without antibiotics and by successive reseeding. Isolates with morphological characteristics resembling those of the genus *Metarhizium* were selected.

We found associated Gram-positive bacteria in the periphery of the hyphae in *Metarhizium* isolates; this interaction persisted even after consecutive reseedings. Conidia obtained from these isolates were decontaminated and washed extensively. These conidia, when observed under the microscope, did not exhibit any external bacterial associations; likewise, when these conidia were inoculated in Sabouraud dextrose medium, the Petri dishes did not show any bacterial colonies.

Two isolates, one from the rhizosphere (ES37) and one from *Phyllopagha* spp larvae (PPH1), were selected for further study. Molecular identification of both isolates was performed by amplifying and sequencing a fragment of the TEF-1α gene [40]. Sequence BLASTn (https://blast.ncbi.nlm.nih.gov/Blast.cgi?PROGRAM=blastn&BLAST_SPEC=GeoBlast&PAGE_TYPE=Blast%E2%80%A6, accessed on 11 November 2025) analysis identified the isolated ES37 strain as *M. robertsii* (99.87% identity), while PPH1 shared the highest similarity with *M. pinghaense* (99.46% identity) (Figure S1).

**Figure 1:**
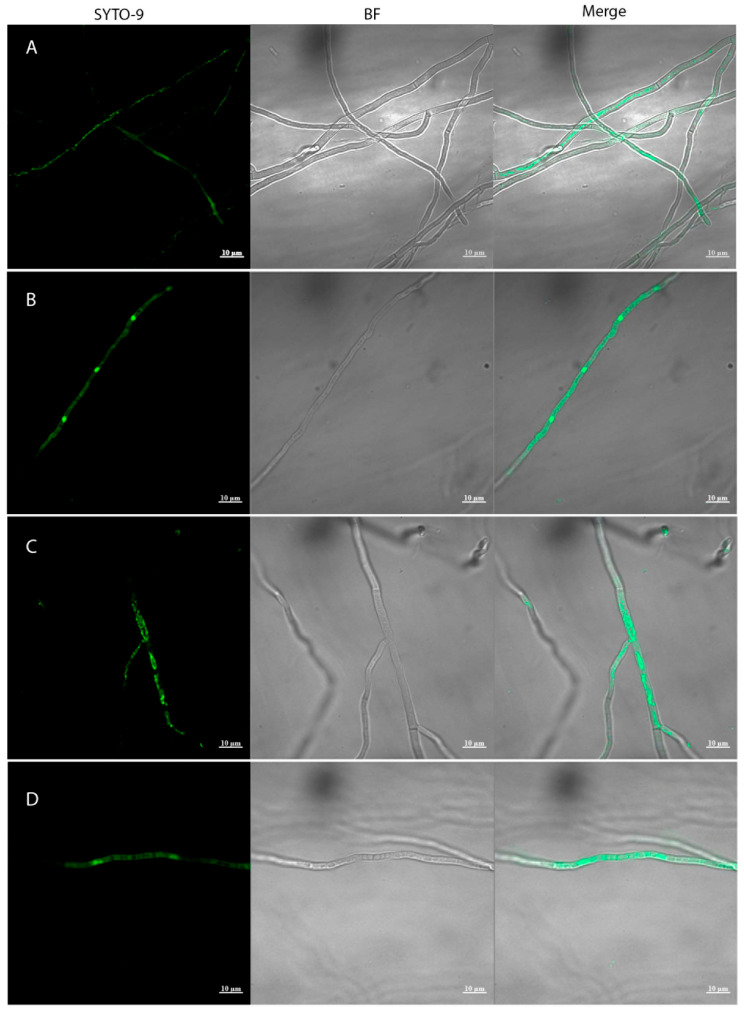
Confocal laser scanning micrographs of *Metarhizium* mycelia stained with SYTO^®^9. *M. robertsii* ES37 (**A**), *M. robertsii* ES37-2 (**B**), *M. pinghaense* PPH1 (**C**), and *M. pinghaense* PPH1-1 (**D**). Samples B and D were overexposed to observe potential EB. Mycelia samples were stained with SYTO^®^9 and analyzed using a laser scanning confocal microscope (ZEISS LSM 700). SYTO^®^9, sample staining. BF, bright field; Merge, bright field, and SYTO^®^9 fluorescence.

The presence of endobacteria in the *M. robertsii* and *M. pinghaense* strains was determined according to the protocol described by Partida-Martinez and Hertweck (2005) [11]. Endobacteria appeared as fluorescent dots within the hyphae (Figure 1A,C). These fluorescent dots were consistently observed in both strains after several successive reseedings. Both strains’ fluorescent dots remained visible after four years of cultivation. To obtain endobacteria-free *Metarhizium*, conidia from the *Metarhizium* strains were cultivated in the presence of antibiotics, as previously reported [11]. Endobacterial loss was confirmed when no amplification products were observed during 16S rDNA PCR amplification (Figure S2). These derivatives were designated ES37-2 and PPH1-1, respectively. Mycelia from these *Metarhizium* derivatives were stained with SYTO^®^9; the green fluorescent dots indicating the presence of endobacteria were not observed, despite overexposure of the samples under the microscope, which showed only nuclear fluorescence (Figure 1B,D). It is essential to note that in some isolates, amplification of the 1.5 Kbp fragment corresponding to the 16S rDNA gene is positive despite the apparent lack of green signals produced by the SYTO^®^9 dye, indicating the presence of EB. Only in isolates *M. robertsii* ES37-2 and *M. pinghaense* PPH1-1, do we obtain a correlation between the lack of green signals and the absence of amplification of the 1.5 Kbp 16S rDNA gene fragment.

### 3.2. Metarhizium Endobacteria Isolation and Identification

To isolate endobacteria, mycelia from *M. robertsii* ES37 and *M. pinghaense* PPH1 were mechanically sheared and subjected to centrifugation. Aliquots of the supernatant were inoculated into five different media. Seven *Bacillus*-like bacterial colonies from the *M. robertsii* ES37 and four colonies from the *M. pinghaense* PPH1 were isolated from YMA medium without Congo red. The 16S rDNA sequence BLASTn analysis revealed that all colonies had the highest similarity with *Bacillus subtilis* (Table S2).

### 3.3. Endobacteria’s Impact on Metarhizium Conidiation

**Figure 2:**
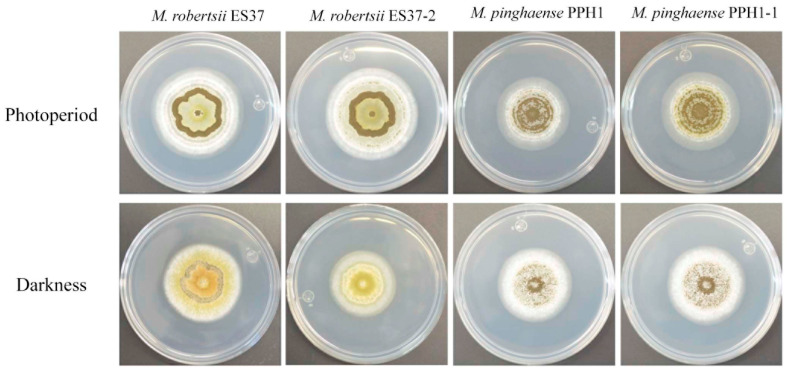
The phenotypes of *Metarhizium* strains in minimal medium. *M. robertsii* ES37 and *M. pinghaense* PPH1 containing EB and their derivatives, EB-free *M. robertsii* ES37-2 and *M. pinghaense* PPH1-1, grow in M-100 medium after 10 days of incubation, both under total darkness and a photoperiod of 16 h light/8 h darkness.

**Figure 3:**
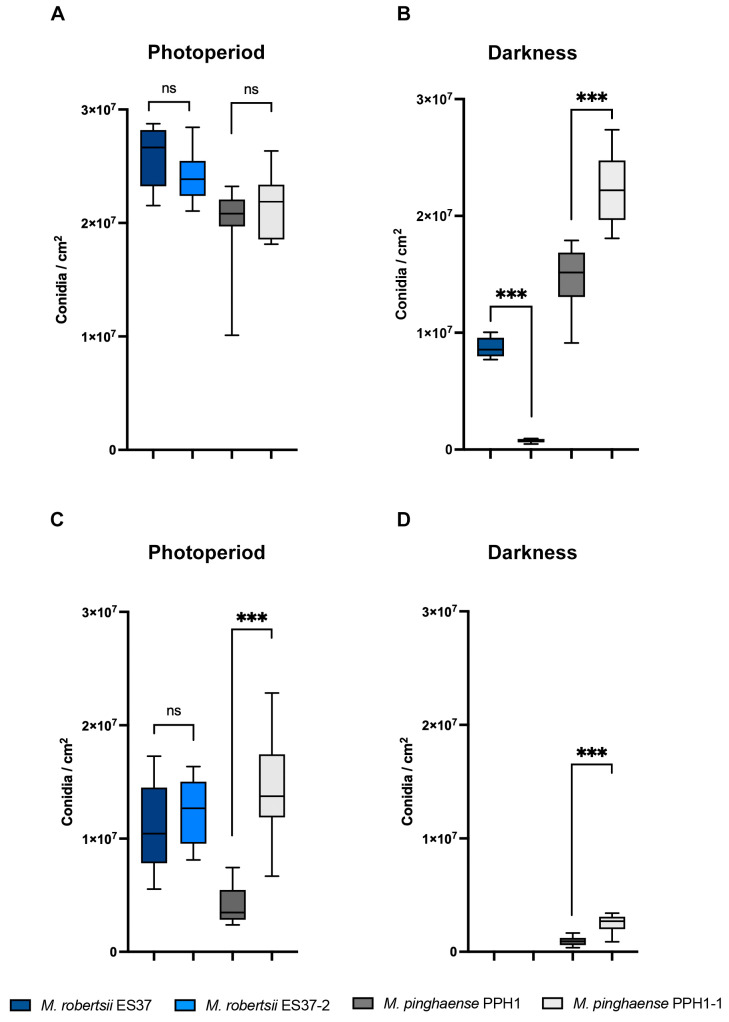
Conidiation rates of *Metarhizium* strains grown in M-100 and PDA media. Conidia production by *M. robertsii* ES37, *M. robertsii* ES37-2, *M. pinghaense* PPH1, and *M. pinghaense* PPH1-1 was quantified after 10 days of incubation in M-100 medium (**A**,**B**) and after 15 days of incubation in PDA medium (**C**,**D**). Petri plates were incubated under photoperiods of 16 h light/8 h darkness (**A**,**C**) and total darkness (**B**,**D**). *t*-test statistical analysis yielded a *p*-value < 0.005. “ns”, no significant difference, *** *p*-value < 0.001.

Since both strains, *M. robertsii* ES37 and *M. pinghaense* PPH1, contain associated bacteria, their germination and conidiation capabilities were assessed to determine whether these endobacteria affected their morphology and growth. The morphology of *Metarhizium* was compared between strains with endobacteria and bacteria-free strains (Figure 2). No significant difference was observed in the radial growth of colonies with endobacteria compared to the bacteria-free colonies. Furthermore, no other morphological changes were noted. The conidia produced by both endobacteria-containing strains and bacteria-free strains, cultured in M-100 and PDA media, were subsequently collected and quantified. Conidial production in M-100 medium in photoperiod showed no significant difference among strains (Figure 3A). However, under dark conditions, the conidiation of *M. robertsii* ES37 with endobacteria was higher than that of the bacteria-free strain (ES37-2). Conversely, the *M. pinghaense* strain with endobacteria (PPH1) exhibited a lower conidiation rate than the bacteria-free strain (PPH1-1) (Figure 3B). In the PDA medium, the conidiation rate of the bacteria-free *M. pinghaense* was higher than that of *M. pinghaense* with endobacteria (Figure 3C,D). No difference was found in the conidiation of the *M. robertsii* strains under photoperiod conditions (Figure 3C), and conidiation was absent under darkness (Figure 3D).

**Figure 4:**
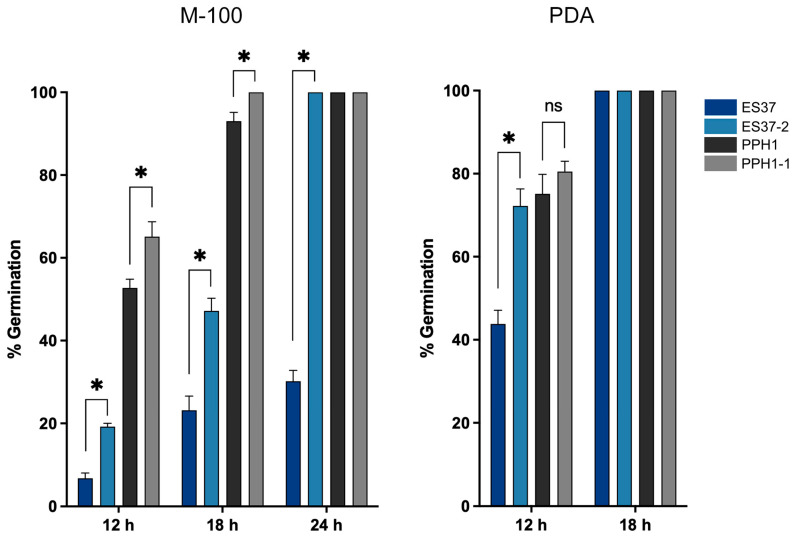
Percentage germination of *Metarhizium* strains. The percentage germination was quantified at 12, 18, and 24 h in M-100 medium, and at 12 and 18 h in PDA medium. “ns”, no significant difference, * *p*-value < 0.001.

The germination of conidia with endobacteria and bacteria-free conidia was analyzed at 12, 18, and 24 h. In the M-100 medium, the germination of bacteria-free strains exceeded that of strains with endobacteria (Figure 4). This difference was particularly pronounced in *M. robertsii* with endobacteria, which exhibited a percentage germination of 6.7% at 12 h; in contrast, bacteria-free *M. robertsii* had a germination percentage of 19.2%. In PDA medium, all strains had surpassed 50% germination within 12 h, except for *M. robertsii* with endobacteria, and by 18 h, all had achieved 100% germination (Figure 4). The results show that the presence of endobacteria generally delays the germination of *Metarhizium*.

### 3.4. Associated Endobacteria Affected Metarhizium Virulence

**Figure 5:**
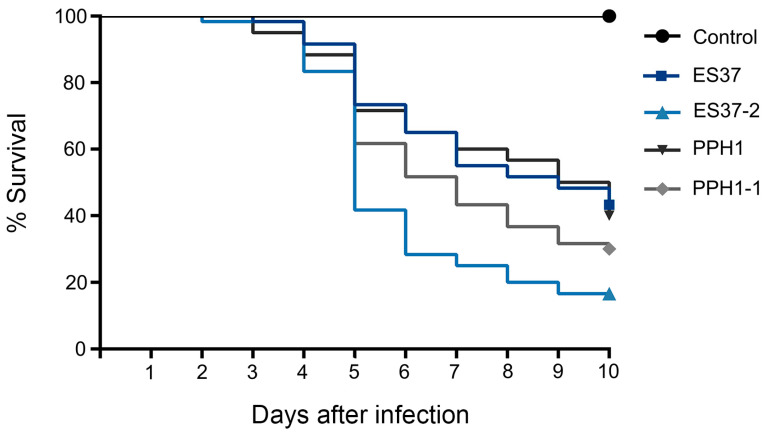
Survival of *G. mellonella* larvae after the application of conidia from *Metarhizium* strains. Conidia (1 × 10^8^ conidia·mL^−1^) from *M. robertsii* ES37, *M. robertsii* ES37-2, *M. pinghaense* PPH1, and *M. pinghaense* PPH1-1 were inoculated into *G. mellonella* larvae, and infection was monitored every 24 h. Control treatments were carried out using 0.01% Triton X-100. The results show the mean percentage survival. The horizontal axis shows days after infection. Statistical analysis: Mantel–Cox (log-rank). X^2^ = 92.71 df = 4; *p*-value < 0.001.

Bioassays were conducted using *Galleria mellonella* larvae to investigate the influence of endobacteria on the virulence of *Metarhizium*. Bacteria-free *M. robertsii* ES37-2 exhibited a higher mortality rate, with over 50% mortality by the fifth day. Both *M. robertsii* ES37 and *M. pinghaense* PPH1 with endobacteria had significantly lower mortality rates compared with the bacteria-free strains (Figure 5). The presence of endobacteria in the two *Metarhizium* isolates resulted in decreased virulence.

**Figure 6:**
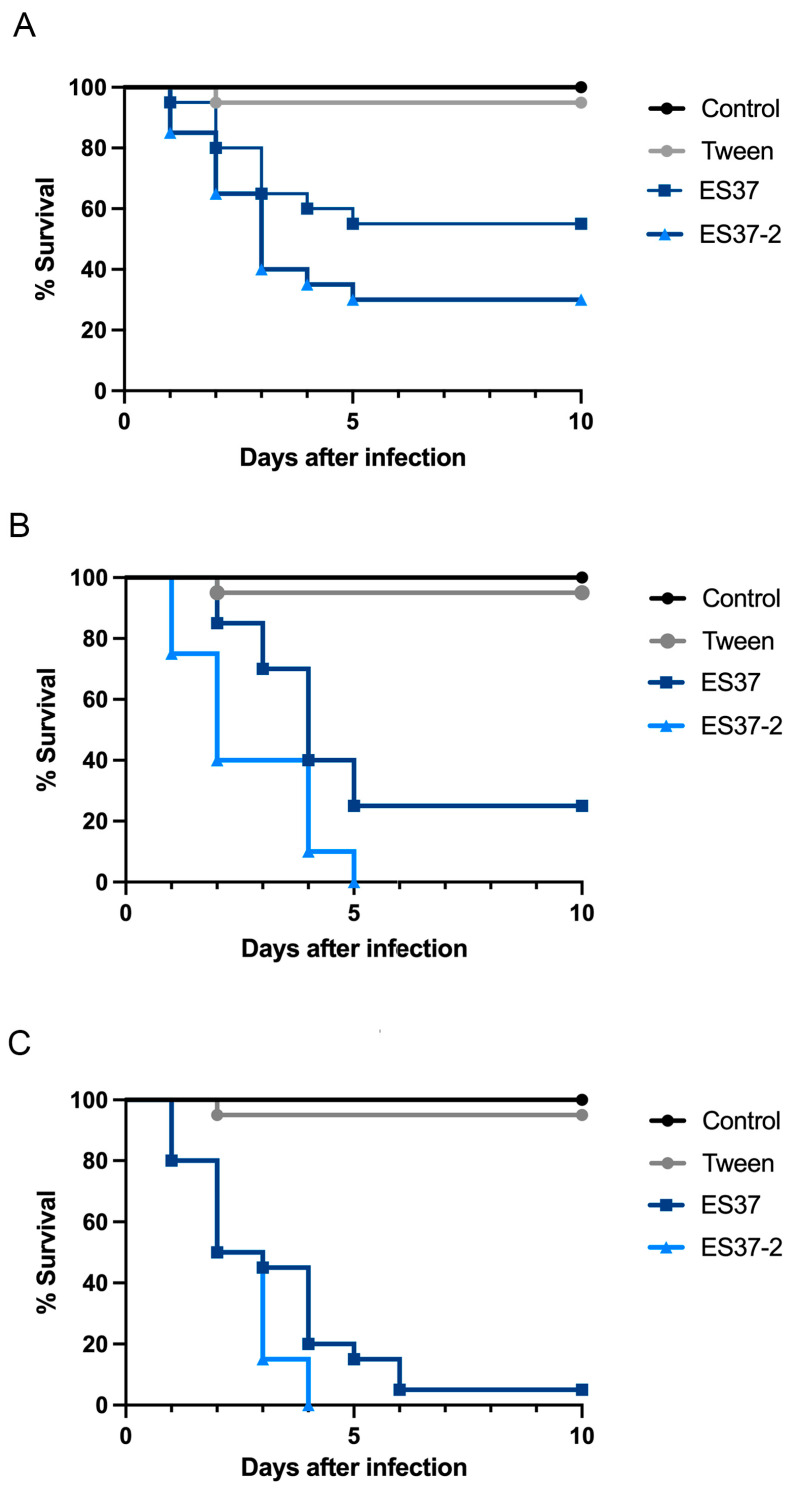
Survival of *T. molitor* larvae after the application of different conidia concentrations of *M. robertsii* ES37 and *M. robertsii* ES37-2 strains: (**A**) 5, (**B**) 50, (**C**) 100 conidia of *M. robertsii* ES37 and ES37-2 strains.

Virulence was measured by injecting *T. molitor* larvae instead of topical infection. Three different conidia concentrations of *M. robertsii* ES37 and ES37-2 strains were analyzed: 5, 50, and 100 (Figure 6). As observed with the topical infection, the presence of endobacteria in *Metarhizium* decreased the virulence, indicating that the presence of EB significantly increased survival.

### 3.5. Genomic Analysis of Associated Endobacteria

**Figure 7:**
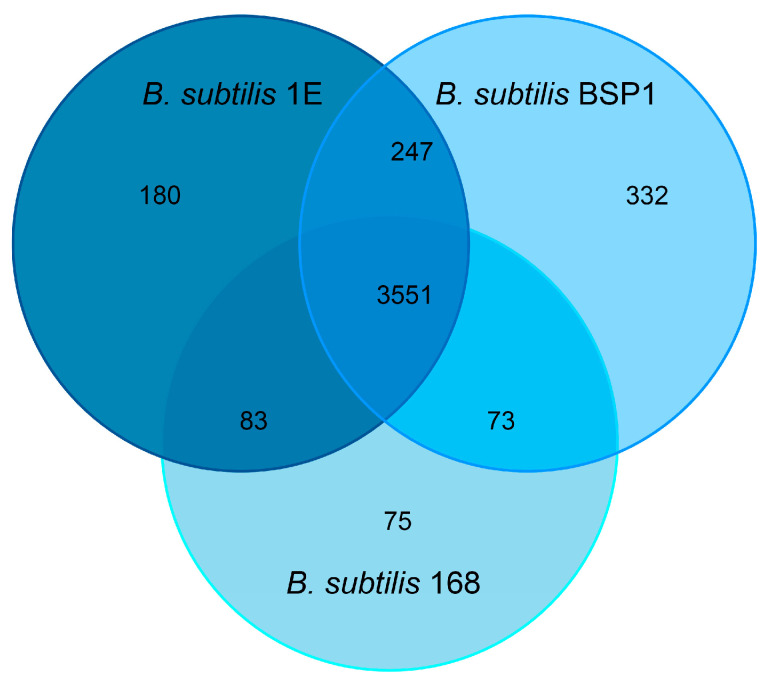
Venn diagram showing the distribution of shared and unique gene clusters among *Bacillus* species: *B. subtilis* 1E, *B. subtilis* BSP1, and *B. subtilis* 168.

**Figure 8:**
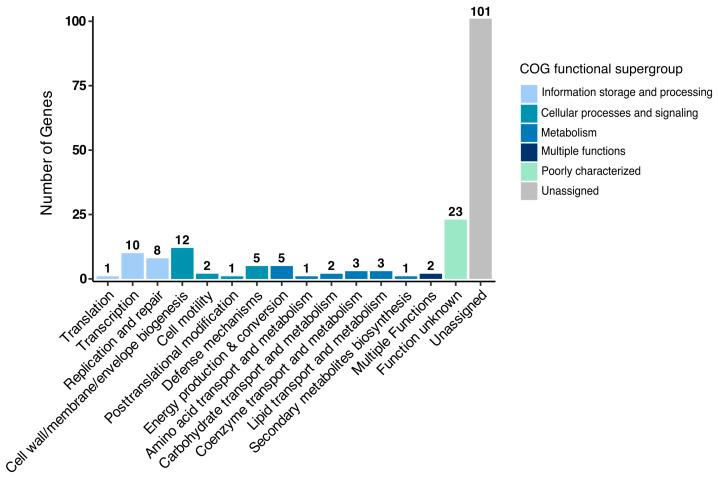
Classification of annotated singleton (genes not found in other species) functions in the Clusters of Orthologous Groups (COG) of *B. subtilis* 1E.

The previous experiments showed the influence of the endobacterium *B. subtilis* on the physiology and virulence of *Metarhizium*, as all the EB isolated in this study were identified as *B. subtilis*. To analyze the genome differences between the *B. subtilis* strain isolated from *Metarhizium* and the *B. subtilis* reference strains, which may explain their presence in *Metarhizium*, the genome of the *B. subtilis* 1E strain, isolated from *M. robertsii* ES37, was sequenced (NCBI accession number CP196825). The genome is 4.1 Mb in size and contains 4242 genes (Table S1). The genome analysis predicted the presence of 86 tRNAs and 30 rRNAs (Table S3). The genome sequence was compared with that of the undomesticated strain *B. subtilis* BSP1 [48] and the reference strain *B. subtilis* 168 (Figure 7). The three genomes share 3551 genes, and the endobacterium *B. subtilis* 1E has 180 genes that are not present in the reference genomes BSP1 and 168 (Table S4). Classification of gene functions in the Database of Clustered Orthologous Groups (COGs) revealed that 101 correspond to an unassigned function, 23 have an unknown function, ten have a transcription function, eight have a replication and repair function, twelve have a cell wall/membrane/biogenesis function, and five have a defense mechanism function (Figure 8). The genes potentially encode proteins with transcription function, including the MerR family transcription factor regulator (OOBLJC_RS00805); members of this family respond to oxidative stress, cellular ion imbalance, toxins, endogenous metabolites, and antibiotics [49,50]. Members of the TetR/AcrT family transcriptional regulator (OOBLJC_RS08765) regulate genes involved in antibiotic production, osmotic stress, efflux pumps, multidrug resistance, metabolic modulation, and pathogenesis [51].

Among the twelve genes that have a cell wall/membrane/biogenesis function, we identified genes involved in the biosynthesis of wall teichoic acids (WTAs), essential components of the Gram-positive bacterial cell wall; the glycerol-3-phosphate cytidylyltransferase (OOBLJC_RS03400), which catalyzes the synthesis of CDP-glycerol; and glycerophosphotransferase (OOBLJC_RS03370; OOBLJC_RS03405; OOBLJC_RS03425), which transfers the glycerolphosphate unit to the growing teichoic acid polymer [52]. The *tarQ* gene (OOBLJC_RS03485) encodes the (poly)ribitol-phosphate teichoic acid beta-D-glucosyltransferase, which attaches β-glucose units to poly(RboP)-WTAs [53]. Analyzing 143 *B. subtilis* genomes revealed that the WTAs’ gene cassettes exhibit a high level of variation, which may be attributed to one or more independent horizontal transfer events during the evolution of *B. subtilis* [54]. Of the 180 genes present in the *B. subtilis* 1E genome, we identified the putative holin-like toxin (OOBLJC_RS08630). One of these proteins was also identified in *B. pumilus*, indicating that it causes toxicity to the host cell by disrupting the membrane [55].

## 4. Discussion

Fungi are successful soil inhabitants due to their plasticity and capacity to respond to adverse or unfavorable conditions [56]. There is growing evidence of fungi, including yeast, and their associated EB, as reported for *Kluyvermyces marxianus* [57] and *Candida albicans* [58,59]. As mentioned previously, more than 90% of land plant species have symbiotic relationships with fungi [18]. Potential bacterial associates have been detected in several fungal isolates [7], and EB have been identified in different phyla of fungi, including Mucormycota, Ascomycota, and Basidiomycota [6]. EB have a distinct influence on fungal development [2,5,60,61]. The impact of EB on fungal resistance to metals has been studied [62]. EB influence fungal sexual reproduction [63] and enhance the production of indole-3-acetic acid [64]. EB (*Bacillus licheniformis*, *Achromobacter xylosoxidans*, and *Stenotrophomonas maltophilia*) in the nematode-trapping fungus *Arthrobotrys musiformis* play an essential role in nitrogen cycling and nematode trap formation [65]. In fungal symbiosis with plants, EB in the fungal host contribute to plant growth and disease resistance to microbial pathogens [5].

Since the *Metarhizium* genus is among the most abundant fungi isolated from soils [19,20], and it has been suggested that many fungi harbor their own microbiomes [4], we investigated the presence of associated endobacteria in native *Metarhizium* strains isolated from the rhizosphere and the insect *Phyllophaga* spp. We found associated Gram-positive bacteria in the periphery of the hyphae in some *Metarhizium* isolates; this interaction persisted even after consecutive reseedings. Conidia were obtained from these isolates, and after surface decontamination and extensive washing, EB were detected using the SYTO^®^9 dye, revealing characteristic green dots, as reported previously in different fungi, including *Metarhizium* [11,39]. These signals persist despite continuous reseeding over four years. These signals were lost when the cells were cultured in the presence of antibiotics through successive reseeding.

To isolate the potential EB, mycelia from *M. robertsii* ES37 and *M. pinghaense* PPH1 were crushed and analyzed in five different media. The bacteria grew only in the Yeast Mannitol Agar (YMA) medium, which is used to cultivate soil microorganisms. Bacterial colonies were isolated from the medium. Molecular analysis identified the colonies as *B. subtilis.* The intracellular occurrence of *Bacillus* has been described in other fungi. In *U. maydis*, *B. pumilus* has been detected in strains isolated from natural sources; these bacteria can fix nitrogen. Studying the interactions between fungi and bacteria in plant rhizoplanes revealed that *Bacillus* was associated as an exobacterium in 80% of the fungi and as a putative endobacterium in 15% of the fungi [66]. In the same study, 80% of the isolated fungi and endobacterial genera potentially involved in the nitrogen cycle were identified [66]. *B. subtilis* was identified as the endobacterium in *Fusarium acuminatum* isolated from the roots of *Spiranthes sinesis* (Orchidaceae) [67]. *B. subtilis* in *Metarhizium* isolates may be a more general phenomenon in soils and insects in different locations. These bacteria may have nitrogen-fixing functions, although they may also employ *Metarhizium* hyphae as a form of dispersal (“fungal highway”), as has been demonstrated in other systems [68,69].

Since *M. robertsii* ES37 was isolated from soil, where complex bacterial communities have been reported [70,71,72], and *M. pinghaense* PPH1 was isolated from *Phyllophaga* spp., where several bacteria have been isolated [73,74], we cannot dismiss the possibility that other EB, besides *B. subtilis*, are present in different *Metarhizium* strains; however, in our *Metarhizium* isolates, we only observed the presence of Gram-positive *Bacillus*-like bacteria.

Our results showed that the presence of endobacteria in *M. robertsii* ES37 and *M. pinghaense* PPH1 delays the conidia germination of *Metarhizium.* This effect is more evident in the *M. robertsii* ES37 strain. In this sense, the impact of bacteria on fungal development is well described; treatment with the *B. subtilis* strain HSY21 has been shown to inhibit the growth and expression of pathogenic genes in *F. oxysporum* [75]. Using the *B. subtilis* strain F62 against *F. oxysporum* showed that the inhibition of mycelial growth occurred primarily through diffusible compounds [76]. Soil isolates of *B. subtilis* exhibited significant fungistatic activity against *M. robertsii* [77].

Similarly, our results showed that endobacteria influence conidia production. Under dark conditions, the conidiation of *M. robertsii* ES37 with endobacteria was higher than that of the bacteria-free strain (ES37-2). This effect was observed in *G. margarita*; the cured line produces only 50% of the spores produced by *G. margarita* containing bacteria [10]. This effect is not observed in the *M. pinghaense* strain PPH1, exhibiting lower conidiation than the bacteria-free strain (PPH1-1). In both strains, *M. robertsii* ES37 and *M. pinghaense* PPH1, the presence of endobacteria decreases virulence against *G. mellonella* (*M. robertsii* ES37 and *M. pinghaense* PPH1) and *T. molitor* (*M. robertsii* ES37). This effect is probably not explained by the delays in conidia germination observed, since *M. robertsii* ES37 and *M. pinghaense* PPH1, in bioassays with *G. mellonella,* have similar insect survival percentages, despite the different influences of endobacteria on conidia germination for each strain. This effect could be the result of the impact of endobacteria on the expression of virulence factors or global transcriptomic changes, as reported in other fungi such as *Rhizopus microsporus*, *G. margarita*, among others [4,78,79].

The influence of *Metarhizium* in the rhizosphere microbiome has not been thoroughly investigated. The community profiles (bacteria and fungi) of the *Phaseolus vulgaris* rhizosphere have been examined following exposure to *M. robertsii* conidia in the presence and absence of *G. mellonella* larvae [80]. The addition of *Metarhizium* conidia increased the quantity of plant growth-promoting organisms, including *Bradyrhizobium*, *Flavobacterium*, *Chaetomium*, and *Trichoderma*. Similarly, the number of members of the genus *Bacillus* increased in the roots following the addition of *Metarhizium* conidia, with this increase being more pronounced in the presence of *G. mellonella* larvae [80]. Recent research suggests that bacteria can exploit fungi for shelter, thereby facilitating survival under unfavorable conditions, as internalization increases bacterial fitness when challenged by abiotic stresses [81].

The presence of *B. subtilis* as an endobacterium in *Metarhizium* suggests a close relationship that can facilitate gene exchange and horizontal gene transfer between the two organisms. It has been reported that *M. robertsii* has 18 genes involved in the infection process, which were potentially acquired through ancient horizontal gene transfer from a bacterium [82]. The authors suggested that the need to degrade insect cuticles served as a selective pressure to retain these genes, as twelve of them are upregulated during penetration, and six have a role in the penetration process [82].

Comparison of the *B. subtilis* 1E genome with those of *B. subtilis* reference strains 168 and BSP1 identified 180 genes that are absent in the reference genomes, 124 of which encode proteins with unassigned or unknown functions. Among the proteins with known function, ten are involved in transcription, eight in replication and repair, twelve in cell wall/membrane/envelope biogenesis, and five in defense mechanisms. A specific function that clearly explains the role of *B. subtilis* 1E in *Metarhizium* has not been identified.

The presence of extracellular bacterial communities or EB in *Metarhizium*, including their influence on behavior in various *Metarhizium* lifestyles, has not been thoroughly studied. Our results demonstrate the inhibitory impact of bacteria on *Metarhizium* growth and virulence capacity; however, there may be mechanisms or conditions under which these bacterial communities provide advantages or benefits to *Metarhizium,* such as through plant interactions in low-nitrogen conditions, in which the presence of EB may provide better survival conditions. For example, we identified 19 genes in the *B. subtilis* 1E genome that encode proteins involved in the nitrogen cycle or in nitrogen metabolism (Table S5), including the putative nitrogen fixation protein *yutI* (OOBLJC_05140), as well as *narG* (nitrate reductase, alpha subunit, OOBLJC_02520), *narH* (nitrate reductase, beta subunit, OOBLJC_02525), and *narI* (nitrate reductase, gamma subunit, OOBLJC_02535), belonging to the *narGHJI* locus, encoding the membrane-bound nitrate reductase, which is significantly induced when *B. subtilis* is undergoing anaerobic growth [83]. Other genes in the *B. subtilis* 1E genome that are involved in nitrate and nitrite respiration and are induced under anaerobic growth conditions include *nark* (nitrate transporter, OOBLJC_02500), *nasD* (Subunit of NADH-dependent nitrite reductase, OOBLJC_19895), and *nasE* (subunit of NADH-dependent nitrite reductase, OOBLJC_19900) [83].

This study presents several opportunities for exploring *Metarhizium* and its applications in agricultural pest control and plant growth promotion—areas that have not been thoroughly investigated to date. Several questions remain to be answered; for example, is the microbiome associated with *Metarhizium* dependent on where it is isolated? Is there a specific association with a particular bacterium? Is there a particular mechanism for bacterial entry, as analyses of lipopeptides have previously suggested? [81]. We plan to address these questions in future work.

## Data Availability

The original contributions presented in this study are included in the article/Supplementary Material. The raw data supporting the conclusions of this article will be made available by the authors on request.

## Supplementary Materials

The following supporting information can be downloaded at https://www.mdpi.com/article/10.3390/jof11110813/s1: Figure S1: Phylogenetic analysis of *Metarhizium* strains; Figure S2: Gel electrophoresis of 16S rDNA fragments; Table S1: Statistics from *B. subtilis* 1E whole-genome sequence analysis; Table S2: Identification of endobacteria cultured from *M. robertsii* ES37 and *M. pinghaense* PPH1; Table S3: Comparative genomic features of *B. subtilis* 1E and the reference *B. subtilis* genomes; Table S4: Comparison of genes in *B. subtilis* 1E and the reference *B. subtilis* genomes; Table S5: Genes present in the *B. subtilis* 1E genome, encoding proteins involved in the nitrogen cycle or in nitrogen metabolism.

## Abbreviations

The following abbreviation is used in this manuscript:

EB	Endobacteria
