# Supplementary material for: Endobacteria Have a Negative Effect on the Virulence of Metarhizium
Source: J Fungi (Basel). 2025 Nov 16;11(11):813. doi: 10.3390/jof11110813 (PMC12653637; doi:10.3390/jof11110813)
Supplement: Supplementary file 1 [file jof-11-00813-s001.zip › Figure S1.pdf]

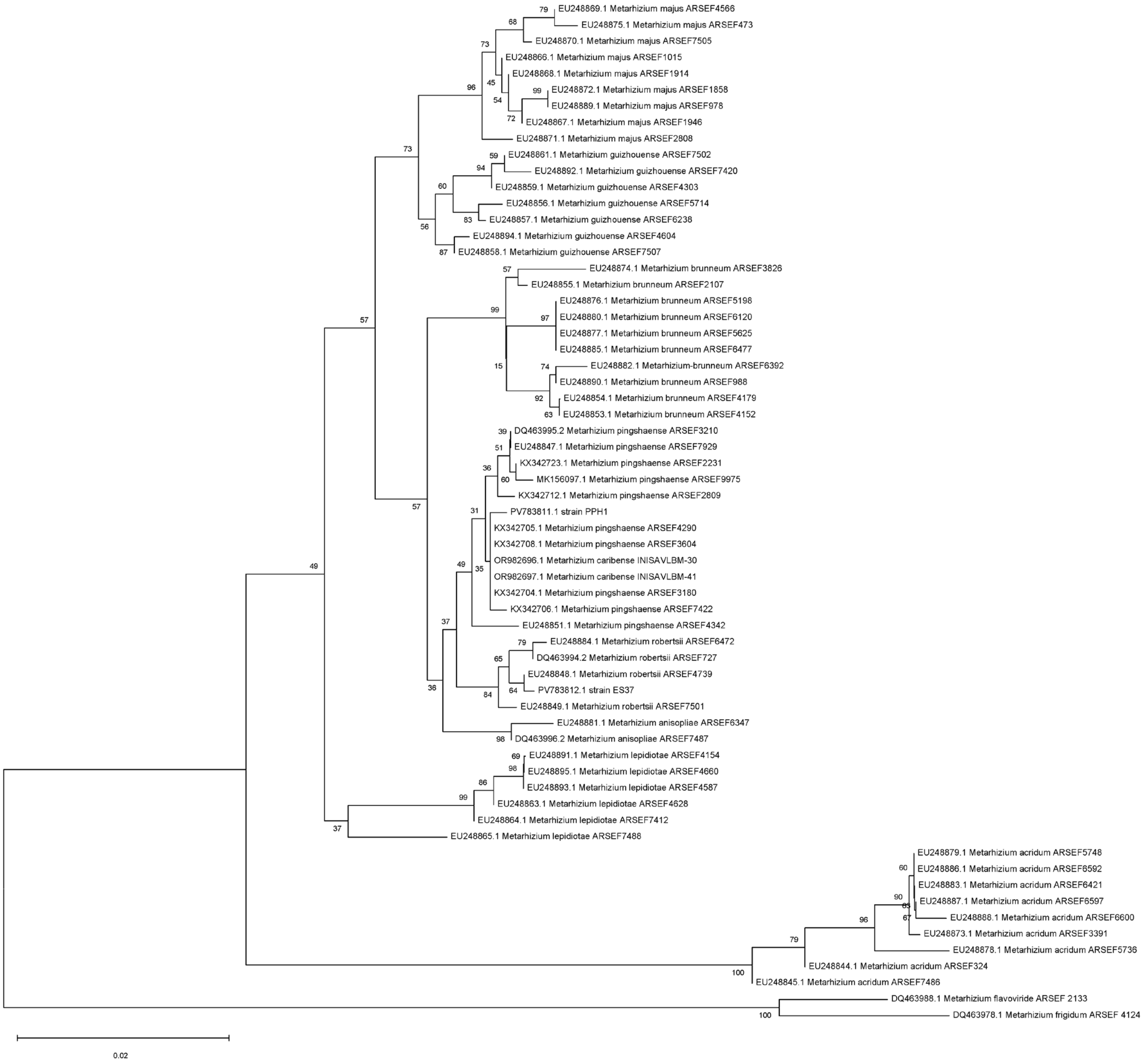

Figure S1. **Phylogenetic analyses of *Metarhizium* strains ES37 and PPH1**. Phylogenetic analyses were performed using the DNA sequence of the 5' region of the TEF-1a gene from 63 *Metarhizium* isolates. The species assignments for strains PPH1 and ES37 are shown, reconstructed using the Neighbor-Joining method with MEGA 12 software (Kumar et al., 2024). A bootstrap test of 1000 replicates was performed. Evolutionary distances and the percentage of replicate trees (bootstrap values) are indicated.

BankIt2968980 *Metarhizium* ES37 (PV783812)
BankIt2964566 *Metarhizium* PPH1(PV783811)

Kumar, S., Stecher, G., Suleski, M., Sanderford, M., Sharma, S., and Tamura, K. (2024). MEGA12: Molecular Evolutionary Genetic Analysis Version 12 for Adaptive and Green Computing. *Molecular Biology and Evolution* 41(12). doi: 10.1093/molbev/msae263.
