# Supplementary material for: Endobacteria Have a Negative Effect on the Virulence of Metarhizium
Source: J Fungi (Basel). 2025 Nov 16;11(11):813. doi: 10.3390/jof11110813 (PMC12653637; doi:10.3390/jof11110813)
Supplement: Supplementary file 1 [file jof-11-00813-s001.zip › Table S1.pdf]

Table S1. Statistics from *B. subtilis* 1E genome sequence

Statistics	*B. subtilis* 1E
Total bp sequenced	873,083,152
Total number of reads	300,451
Longest read	86,623
Raw coverage	210x
Assembled coverage	103x
Genome size (Mb)	4.1
Number of genes annotated	4,242
