# Supplementary material for: Endobacteria Have a Negative Effect on the Virulence of Metarhizium
Source: J Fungi (Basel). 2025 Nov 16;11(11):813. doi: 10.3390/jof11110813 (PMC12653637; doi:10.3390/jof11110813)
Supplement: Supplementary file 1 [file jof-11-00813-s001.zip › Table S2.pdf]

Table S2. Endobacteria cultured from *M. robertsii* ES37 and *M. pinghaense* PPH1

Strain	Isolates	Accession Number (NCBI)	Specie	% Identity	% Cover	Accession number (GenBank)
M. robertsii ES37	4A	PV770101	*Bacillus subtilis* subsp. subtilis str. 168	99.63	99	8BUU_a
	2B	PV770096	*Bacillus subtilis* subsp. *subtilis* str. 168	99.71	99	8BUU_a
	1C	PV770103	*Bacillus subtilis* subsp. *subtilis* str. 168	99.71	100	8BUU_a
	4C	PV770099	*Bacillus subtilis* subsp. *subtilis* str. 168	99.63	99	8BUU_a
	1E	PV770095	*Bacillus subtilis* subsp. *subtilis* str. 168	99.78	99	8BUU_a
	I-2	PV770102	*Bacillus subtilis* subsp. *subtilis* str. 168	99.71	99	8BUU_a
	I-4	PV770104	*Bacillus subtilis* subsp. *subtilis* str. 168	99.56|	99	8BUU_a
M. pinghaense PPH1	1B	PV770094	*Bacillus subtilis* subsp. *subtilis* str. 168	99.58	99	8BUU_a
	4B	PV770097	*Bacillus subtilis* subsp. *subtilis* str. 168	99.78	99	8BUU_a
	2C	PV770100	*Bacillus subtilis* subsp. *subtilis* str. 168	99.63	99	8BUU_a
	4D	PV770098	*Bacillus subtilis* subsp. *subtilis* str. 168	99.63	99	8BUU_a
