# Supplementary material for: Endobacteria Have a Negative Effect on the Virulence of Metarhizium
Source: J Fungi (Basel). 2025 Nov 16;11(11):813. doi: 10.3390/jof11110813 (PMC12653637; doi:10.3390/jof11110813)
Supplement: Supplementary file 1 [file jof-11-00813-s001.zip › Table S3.pdf]

Table S3. Comparative genomic features of *B. subtilis* IE and the reference *B. subtilis* genomes.

Feature	*B. subtilis* 1E	*B. subtilis* BSP1	*B. subtilis* 168
tRNAs	87	84	86
rRNAs	30	30	30
ncRNAs	28	2	118
CDSs	4,096	4,049	4,237
Pseudogenes	16	253	88
Hypotheticals	161	276	347
Genes	4,242	4,168	4,546
Proteins	4,105	3,796	4,243
