# Supplementary figures and images for: Endobacteria Have a Negative Effect on the Virulence of Metarhizium
Source: J Fungi (Basel). 2025 Nov 16;11(11):813. doi: 10.3390/jof11110813 (PMC12653637; doi:10.3390/jof11110813)

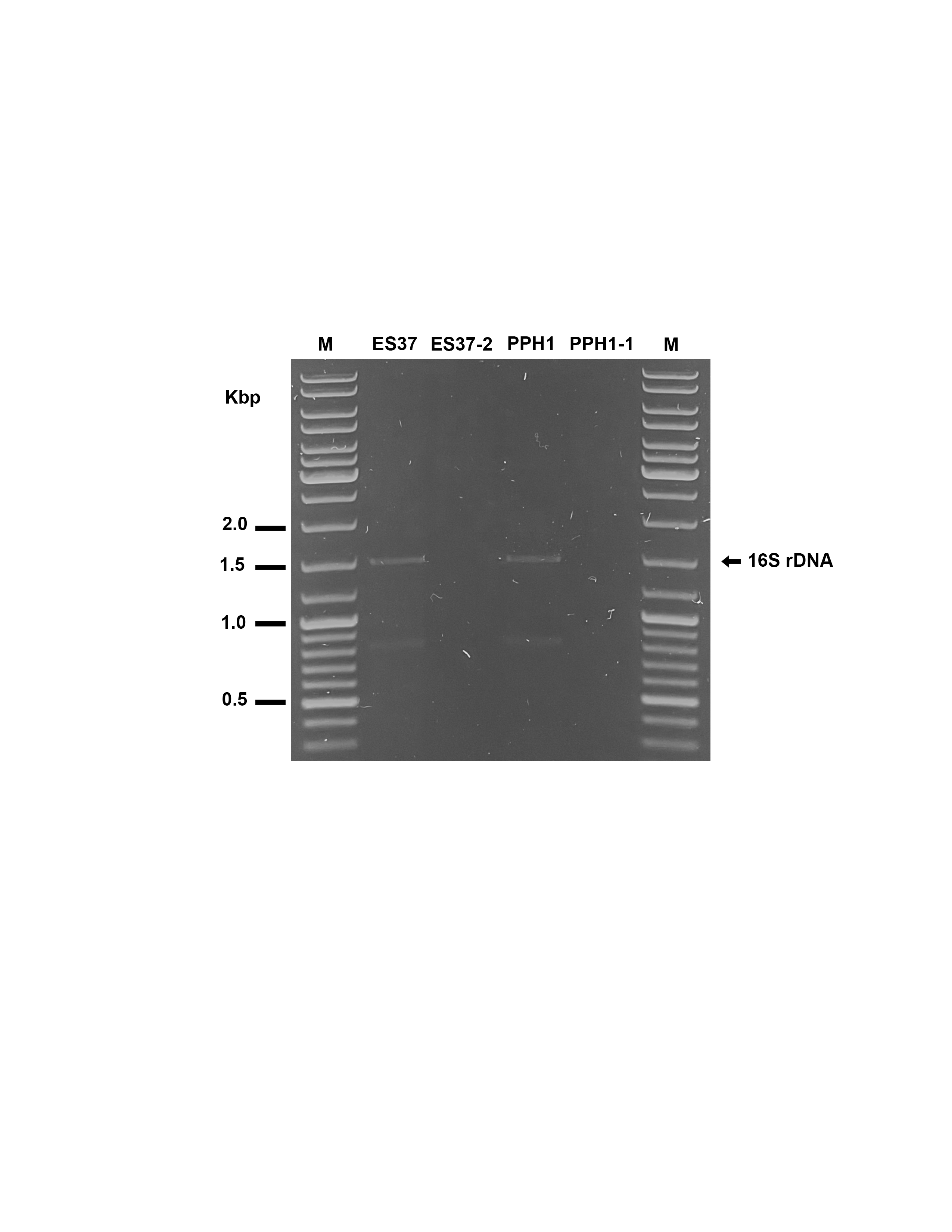

Supplement: Supplementary file 1 [file jof-11-00813-s001.zip › Figure S2.tif]
